# Current and Future Perspectives of Adjuvant Therapy for Resected Colorectal Liver Metastases

**DOI:** 10.3390/cancers18081188

**Published:** 2026-04-08

**Authors:** Kozo Kataoka, Kei Kimura, Ayako Imada, Kazuma Ito, Zhenxin Rao, Yuko Fukumoto, Jihyung Song, Yuki Horio, Ryuichi Kuwahara, Motoi Uchino, Takayuki Yoshino, Eiji Oki, Yukihide Kanemitsu, Masataka Ikeda

**Affiliations:** 1 Division of Lower GI Surgery, Department of Gastroenterological Surgery, Hyogo Medical University, Nishinomiya 663-8131, Japan; 2 Division of Inflammatory Bowel Disease Surgery, Department of Gastroenterological Surgery, Hyogo Medical University, Nishinomiya 663-8131, Japan; 3 Department of Gastrointestinal Oncology, National Cancer Center Hospital East, Kashiwa 277-8577, Japan; 4 Department of Advanced Medicine and Innovative Technology, Kyushu University Hospital, Fukuoka 812-8582, Japan; 5 Department of Colorectal Surgery, National Cancer Center Hospital, Tokyo 104-0045, Japan

**Keywords:** colorectal liver metastases, adjuvant therapy, circulating tumor DNA

## Abstract

The liver is the most common site of metastatic disease in patients with colorectal cancer. However, the multidisciplinary management of colorectal liver metastases (CLMs) remains suboptimal. Over the past several decades, numerous randomized trials have evaluated the efficacy of adjuvant chemotherapy following CLM resection, revealing improvements in disease-free survival. Nevertheless, these studies have not consistently demonstrated benefits in overall survival, resulting in controversy with regard to the role of routine postoperative chemotherapy. Circulating tumor DNA (ctDNA) has recently emerged as a promising biomarker for detecting molecular residual disease after surgery. Multiple studies have consistently shown that postoperative ctDNA positivity is strongly associated with inferior recurrence-free survival and overall survival in patients with colorectal cancer. In addition to its prognostic value, ctDNA may also assist in guiding postoperative therapeutic decisions. In prospective observational studies of CLM, adjuvant chemotherapy provided potential clinical benefits primarily in patients with ctDNA-positive disease, whereas limited benefits were observed in ctDNA-negative patients. These findings suggest that ctDNA-based detection of molecular residual disease may aid in developing a framework for risk-adapted postoperative management after CLM resection. However, several challenges remain, including the identification of an optimal treatment regimen for ctDNA-positive patients and the improvement of ctDNA assay sensitivity. Ongoing biomarker-driven clinical trials may clarify whether ctDNA-guided strategies can improve patient selection and clinical outcomes following curative resection of CLM.

## Simple Summary

Colorectal liver metastases (CLMs) are the most common form of distant metastasis in patients with colorectal cancer (CRC). However, the multidisciplinary management of CLM remains suboptimal. Several randomized trials have investigated the role of adjuvant chemotherapy after CLM resection, showing improvements in recurrence-related outcomes. Nevertheless, the available studies do not consistently demonstrate a clear benefit in overall survival, and the optimal postoperative management strategy is currently uncertain. Circulating tumor DNA (ctDNA) has recently emerged as a promising biomarker for detecting minimal residual disease postsurgery. Studies have shown that patients with detectable ctDNA after surgery have a significantly higher risk of recurrence and worse survival outcomes. Importantly, recent evidence indicates that adjuvant chemotherapy may primarily benefit patients with ctDNA-positive disease. Therefore, ctDNA-guided strategies may assist in personalizing postoperative treatment and improve patient selection following CLM resection.

## 1. Introduction

Colorectal liver metastases (CLMs) develop in approximately one-third of patients with colorectal cancer (CRC) during the course of their disease [1,2]. Surgical resection remains the cornerstone of potentially curative treatment; however, long-term cures are achieved in only a subset of patients, and disease recurrence occurs in the majority, even after seemingly curative hepatectomy. Consequently, the optimal postoperative management strategy following CLM resection remains one of the most persistent and unresolved issues in the multidisciplinary management of metastatic CRC. Over the past two decades, considerable efforts have been devoted to evaluating the role of adjuvant chemotherapy (ACT) after curative-intent liver metastasectomy [3,4,5,6,7,8]. Although several randomized trials have demonstrated improvements in disease-free survival (DFS) or progression-free survival (PFS), a clear benefit in overall survival (OS) has not been consistently observed. These findings have generated ongoing debate regarding the routine use of ACT in this setting and highlight the need for more refined strategies to identify patients who are most likely to benefit from postoperative systemic therapy.

Recent advances in molecular diagnostics have identified circulating tumor DNA (ctDNA) as a promising biomarker for the detection of molecular residual disease (MRD) [9,10,11,12]. By providing a real-time measure of tumor burden at the molecular level, ctDNA has emerged as a powerful prognostic marker and a potential tool to guide postoperative therapeutic decisions. This biomarker-driven approach offers an opportunity to move beyond conventional clinicopathologic risk stratification toward more individualized treatment strategies.

In this review, we summarize the available evidence regarding ACT following resection of CLM and discuss the limitations of current treatment strategies. We further highlight the emerging role of ctDNA-based MRD detection as a framework for risk stratification and treatment selection, and we explore future perspectives for biomarker-driven postoperative management in patients undergoing curative resection of CLM. The literature discussed in this review was identified through a structured search of PubMed and major conference proceedings, with a focus on key prospective studies, randomized trials, and relevant observational analyses in the field of colorectal liver metastases and ctDNA. Priority was given to studies with clinical relevance and methodological rigor.

## 2. Current Status of Systemic Therapy in Resected CLM

As noted above, numerous phase III randomized trials conducted over the past two decades have investigated the role of ACT after curative-intent resection of CLM. The results of these studies demonstrated that ACT consistently improved DFS, PFS, or recurrence-free survival (RFS); however, they did not show a definitive benefit in OS [3,4,5,6,7,8]. A brief summary of pivotal studies of ACT in resectable CLM is provided in Table 1. Although the proportion of patients with synchronous disease differed across the studies, the metastatic burden was broadly comparable, with a median of one liver metastasis in all trials, except for one.

The FFCD (Fédération Francophone de Cancérologie Digestive) trial compared fluoropyrimidine-based chemotherapy with surgery alone. Fluorouracil/leucovorin (5-FU/LV) was associated with a significant improvement in DFS compared with surgery alone, although no statistically significant advantage in OS was observed [3]. Similarly, a pooled analysis of the FFCD and ENG (European Organisation for Research and Treatment of Cancer [EORTC]/National Cancer Institute of Canada Clinical Trials Group/Gruppo Italiano di Valutazione Interventi in Oncologia) trials showed that adjuvant 5-fluorouracil/leucovorin was associated with a trend toward improved DFS compared with surgery alone, without corresponding OS benefits [4]. A randomized controlled study comparing postoperative uracil–tegafur/leucovorin with surgery alone (University Hospital Medical Information Network [UMIN] C000000013) showed the benefit of adjuvant uracil–tegafur/leucovorin in RFS but not in OS [7]. Moreover, Ychou et al. reported that adjuvant 5-fluorouracil/folinic acid with irinotecan did not improve DFS or OS compared with fluoropyrimidine-based therapy [5]. The Japan Clinical Oncology Group (JCOG0603) compared postoperative modified FOLFOX6 (mFOLFOX6) with observation in resected CLM [8]. Similarly, the findings demonstrated a significant DFS advantage in the mFOLFOX6 arm (HR: 0.67, *p* = 0.006), but no OS benefits were observed (HR: 1.25, *p* = 0.42). This lack of improvement in OS was confirmed in an updated analysis [13].

In contrast to postoperative adjuvant trials, perioperative chemotherapy has also been evaluated in patients with resectable CLM. The EORTC 40983 trial investigated perioperative fluorouracil, leucovorin, and oxaliplatin (FOLFOX) compared with surgery alone [6,14]. Perioperative FOLFOX was associated with a significant improvement in progression-free survival in eligible patients (HR, 0.78; *p* = 0.035), although this did not translate into overall survival benefits (HR, 0.88; *p* = 0.34).

More recently, an individual patient data meta-analysis of randomized phase III trials evaluating ACT in resectable CLM was performed, including EORTC 40983/EPOC, FFCD-ACHBTH-AURC 9002 (FFCD-Association de Chirurgie Hépato-Biliaire et de Transplantation Hépatique-Association Universitaire de Recherche en Chirurgie), ENG, and UMIN C000000013, but excluding JCOG0603 [15]. In the overall intention-to-treat population (N = 821), systemic chemotherapy significantly improved PFS (HR: 0.79, 95% confidence interval [CI]: 0.67–0.93; *p* = 0.004) and was associated with a marginal OS benefit (HR: 0.82, 95% CI: 0.68–1.00; *p* = 0.048). However, after adjustment for prognostic factors in multivariable analyses, the benefit of systemic chemotherapy remained significant for PFS (*p* = 0.0002) but was no longer evident for OS (*p* = 0.102). Notably, a significant interaction between systemic chemotherapy and synchronicity was observed for PFS in the intention-to-treat population (*p* = 0.027). Taken together, these findings suggest that routine administration of ACT cannot be universally recommended after CLM resection.

This uncertainty is also reflected in current clinical practice guidelines. The National Comprehensive Cancer Network Guidelines list capecitabine plus oxaliplatin (CAPOX), mFOLFOX6, and fluoropyrimidine-based therapy as postoperative options but do not mandate treatment [16]. Similarly, the European Society for Medical Oncology Guidelines state that postoperative chemotherapy may be considered, although it cannot be regarded as a standard of care because of the limited evidence from randomized studies supporting its use [17]. The Japanese Society for Cancer of the Colon and Rectum Guidelines provide only a weak recommendation for postoperative therapy [18].

The reasons for the discrepancy between DFS and OS remain unclear. A possible explanation is chemotherapy-associated liver injury, including sinusoidal obstruction syndrome and steatohepatitis. Such treatment-related hepatic changes may reduce the sensitivity of computed tomography (CT) for detecting small residual or recurrent liver metastases [19]. In JCOG0603, liver recurrence was more frequent in the observation group than in the mFOLFOX6 group (61% vs. 47%, respectively), which may partly support this hypothesis. In recent years, magnetic resonance imaging (MRI) using hepatocyte-specific gadolinium-based contrast agents has shown superior diagnostic performance for detecting liver metastases compared to a CT scan alone. In the CAMINO trial, contrast-enhanced liver MRI altered the planned treatment strategy in approximately one-third of patients with CLM [20]. Similarly, in the DREAM trial (EORTC1527/JCOG1609INT), one-third of disappearing liver metastases identified only via CT remained detectable on MRI [21]. These findings suggest that future clinical trials should incorporate contrast-enhanced MRI in addition to contrast-enhanced CT when defining eligibility and evaluating patterns of recurrence.

Another possible explanation is adjuvant therapy-related shortening of survival (ATRESS) [22]. This hypothesis proposes that postoperative chemotherapy may promote treatment resistance in residual tumor clones. However, supporting evidence for this concept remains limited, and further investigation is warranted.

From a statistical perspective, the surrogacy of DFS or RFS for OS has not been firmly established in resected CLM. Ecker et al. reported only a weak correlation between RFS and OS in both a meta-analysis of adjuvant therapy trials and retrospective institutional data spanning 30 years [23]. By contrast, an analysis based on a Japanese nationwide cohort suggested a moderate, although not statistically significant, correlation between RFS and OS [24]. Thus, RFS cannot currently be regarded as a validated surrogate endpoint for OS in future trials involving surgically resected CLM. In addition, differences in post-recurrence management may also contribute to the observed discrepancy between recurrence-related endpoints and OS. The availability of effective salvage treatments, including repeat resection or local ablative therapies, as well as advances in systemic therapy, may attenuate differences in OS despite earlier recurrence.

## 3. ctDNA Biomarker-Driven Treatment Strategy

### 3.1. Prognostic Role of ctDNA in MRD Detection

The presence of cell-free DNA in blood samples was first described in 1948 [25]. Technological advances in recent decades have enabled the detection of ctDNA, a tumor-derived fraction of cell-free DNA released into the bloodstream. Following its release, ctDNA is rapidly cleared from the circulation, with an estimated half-life of approximately 2 h. Initially, ctDNA analysis was primarily used to identify specific tumor-associated genetic alterations. More recently, however, ctDNA has emerged as a highly promising biomarker for detecting MRD, refining postoperative risk stratification, and guiding adjuvant treatment decisions [9,10,11,12,26,27,28,29,30]. A summary of studies evaluating ctDNA as a marker of MRD is shown in Table 2. The results of these reports have been remarkably consistent, revealing that patients with postoperative ctDNA positivity experience substantially worse RFS and OS than those with postoperative ctDNA negativity. In the GALAXY study, the largest ctDNA platform study of CRC, postoperative ctDNA positivity was strongly associated with inferior DFS (HR 11.99, 95% CI 10.02–14.35) and OS (HR 9.68, 95% CI 6.33–14.82) [29]. These findings indicate that ctDNA is not only a powerful prognostic marker but also a potentially useful tool for postoperative treatment stratification. The clinical utility of ctDNA-guided management was further highlighted in the DYNAMIC trial. The study included 455 patients with stage II CRC, who were randomly assigned to a ctDNA-guided management group or a standard management group [31]. The ctDNA-guided strategy reduced the use of ACT without compromising RFS, supporting the feasibility of biomarker-guided postoperative treatment. Similarly, in the GALAXY study, patients with stage II/III disease who were ctDNA-positive derived benefit from ACT after adjustment for clinicopathological factors, whereas no such benefits were observed in ctDNA-negative patients. These results underscore the importance of ctDNA as a prognostic biomarker, as well as a potential predictive biomarker for ACT benefits. Further investigation is warranted.

### 3.2. Available Evidence Regarding ctDNA in CLM

Only two prospective studies have evaluated the association between ACT and ctDNA status in resected CLM (Table 3). In a subgroup analysis of patients with CLM who underwent upfront surgery in the GALAXY study, ACT was associated with a marked DFS benefit in the MRD-positive group (adjusted HR 0.07, 95% CI 0.02–0.26; *p* < 0.0001), whereas no DFS benefits were observed in the MRD-negative group (adjusted HR 0.68, 95% CI 0.29–1.58; *p* = 0.371) [32]. Of note, OS data according to ctDNA status were not reported in that analysis.

Comparable findings were reported in the prospective PKUCRLM-01 cohort [33]. In this study, approximately two-thirds of patients with CLM received preoperative chemotherapy. ACT conferred a significant RFS benefit in patients who were ctDNA-positive at 1 month after surgery (HR 0.228, 95% CI 0.116–0.446; *p* < 0.0001), whereas no significant RFS benefits were observed in the ctDNA-negative subgroup (*p* = 0.39). Importantly, this study also reported OS outcomes according to ctDNA status. The OS results were consistent with the RFS findings: ACT improved OS in ctDNA-positive patients (HR 0.357, 95% CI 0.133–0.963; *p* = 0.034), while no OS benefits were observed in ctDNA-negative patients (*p* = 0.19).

Collectively, these studies support the role of ctDNA as a prognostic biomarker for recurrence risk. However, its predictive value for guiding postoperative treatment decisions remains to be fully established, particularly in patients with resected CLM. The evidence suggesting a benefit of ACT in ctDNA-positive patients is based on a limited number of studies, including observational analyses and datasets with incomplete OS information; therefore, it should be interpreted with caution. Although ctDNA appears to have markedly higher prognostic value than conventional clinicopathological factors, combining ctDNA status with established risk features may further refine risk stratification. Indeed, even among patients with CLM who are MRD-negative, ACT may still be associated with improved DFS in those with other adverse prognostic features, such as multiple liver metastases or synchronous disease. In the CLM subgroup of the GALAXY study, a marginal benefit of ACT was observed in patients with synchronous CLM and MRD positivity (HR 0.21, 95% CI 0.04–1.08; *p* = 0.062). Further prospective validation is required before ctDNA-guided treatment strategies can be fully incorporated into routine clinical practice. Despite its strong prognostic value, ctDNA-based MRD detection has inherent limitations. In particular, false-negative results may occur in patients with low-volume disease or metastases at sites with limited ctDNA shedding, such as the lung or peritoneum [34,35]. This limitation is especially relevant in CLM, where patterns of recurrence may differ according to ctDNA status. In ctDNA-positive patients, liver metastases are more frequently observed, whereas in ctDNA-negative patients, recurrence more commonly involves the lung or peritoneum. Taken together, these findings highlight both the strengths and limitations of ctDNA-based MRD detection in CLM. While ctDNA provides a powerful tool for identifying patients at high risk of recurrence, its performance appears to be influenced by tumor biology, metastatic burden, and the anatomical site of disease. Therefore, ctDNA negativity should not be interpreted as the complete absence of residual disease. An integrated approach combining ctDNA with conventional clinicopathological risk factors and high-quality imaging modalities may represent the most appropriate strategy for postoperative risk stratification in current clinical practice.

### 3.3. Future Perspective of ctDNA-Based Treatment Strategies

The clinical implementation of ctDNA-guided strategies requires careful consideration of several practical aspects. The timing of postoperative sampling is particularly important, as early measurements may be influenced by background cell-free DNA released from surgical tissue injury, potentially reducing specificity. Therefore, an appropriate interval after surgery is generally recommended before initial ctDNA assessment.

Serial monitoring may further enhance clinical utility by enabling dynamic assessment of MRD status and earlier detection of recurrence. In addition, ctDNA detectability may vary according to tumor burden and the site of recurrence. Patients with low-volume disease or metastases at sites such as the lung or peritoneum may exhibit lower ctDNA shedding, which could lead to false-negative results. These biological and technical factors should be considered when interpreting ctDNA results in the postoperative setting. In addition, the standardization of assay platforms and the definition of clinically meaningful endpoints, such as ctDNA clearance, remain important challenges. Furthermore, the cost-effectiveness and accessibility of ctDNA testing will be important factors influencing its adoption in routine clinical practice across different healthcare systems.

Although multiple studies have indicated that ACT may benefit patients with MRD-positive disease [27,28,29,30,31,34], several key issues remain unresolved, including the optimal adjuvant regimen for ctDNA-positive CRC and the sensitivity of ctDNA assays. Importantly, ongoing trials are not only evaluating treatment efficacy but are also expected to provide critical insights into the clinical utility of ctDNA-guided strategies. In addition, the harmonization of assay methodologies and standardization of reporting criteria will be essential to facilitate comparison across studies and to enable broader implementation in clinical practice.

### 3.4. Optimal Adjuvant Regimen for ctDNA-Positive CRC

At present, the ALTAIR trial is the only randomized controlled study to have specifically evaluated treatment in postoperative ctDNA-positive CRC [36]. In this study, trifluridine/tipiracil (FTD/TPI) was compared with placebo in patients with ctDNA-positive CRC after curative-intent surgery. The median DFS was 9.30 and 5.55 months in the FTD/TPI group (n = 122) and the placebo group (n = 121), respectively; however, the study did not meet its primary endpoint (HR: 0.79, 95% CI: 0.60–1.05; *p* = 0.107). In a subgroup analysis of patients with stage IV disease, FTD/TPI benefits were suggested (HR: 0.53, *p* = 0.012). Several ongoing trials are currently testing whether more intensive postoperative treatment strategies, including FOLFOXIRI-based regimens, may improve outcomes in MRD-positive CRC after curative resection. These trials include CIRCULATE-US (NCT05174169), AFFORD (NCT05427669), and CLAUDIA (NCT05534087) [37]. Preliminary results from DYNAMIC-III have recently been reported [38]. In this trial, ctDNA-guided management was compared with standard management in patients with resected stage III colon cancer. Within the ctDNA-guided arm, ctDNA-negative patients received de-escalated ACT, whereas ctDNA-positive patients received escalated ACT. A post hoc analysis comparing FOLFOXIRI with doublet regimens (mFOLFOX6/CAPOX) in ctDNA-positive patients found no improvement in ctDNA clearance or RFS with FOLFOXIRI. Notably, Kaplan–Meier curves for RFS numerically favored the doublet regimen arm, with 3-year RFS rates of 51% and 47% for mFOLFOX6/CAPOX and FOLFOXIRI, respectively. The ctDNA clearance rates were also similar (62% vs. 60%, respectively). Consistent observations were recorded in the non-randomized phase II FANTASTIC study, which evaluated mFOLFOXIRI after resection of metastatic CRC [39]. Although the mFOLFOXIRI group showed a numerically favorable trend for RFS compared with the standard-of-care group, the ctDNA clearance rate was only 33%.

Taken together, these findings suggest that intensification of cytotoxic chemotherapy alone is unlikely to overcome the biology underlying MRD-driven recurrence. This supports a conceptual shift from indiscriminate treatment escalation toward biomarker-informed strategies. Rather than simply increasing the intensity of chemotherapy, future approaches for ctDNA-positive disease may require biologically selected treatment, including the incorporation of targeted agents such as anti-vascular endothelial growth factor (anti-VEGF) or anti-epidermal growth factor receptor (anti-EGFR) therapies. In Japan, an ongoing randomized phase II trial is comparing mFOLFOXIRI plus bevacizumab with mFOLFOX6 in resected oligometastatic CRC [40].

### 3.5. Improving ctDNA Assay Performance for MRD Detection

ctDNA assays are heterogeneous in their design, which may influence clinical interpretation [41,42]. Tumor-informed assays, such as personalized mPCR-NGS approaches, are designed based on patient-specific mutations identified from tumor tissue and generally offer higher sensitivity for MRD detection [26]. In contrast, tumor-agnostic (plasma-only) assays rely on predefined panels or epigenomic signatures and may provide broader applicability without the need for tumor tissue, although potentially at the cost of reduced sensitivity in low-burden disease [43,44]. Furthermore, emerging methylation-based and fragmentomics-based approaches have shown promise in improving detection sensitivity, particularly in cases with low ctDNA shedding. These methodological differences should be taken into account when interpreting results across studies, as they may partly explain the variability in reported ctDNA positivity rates and prognostic performance [45].

Importantly, these technical considerations have direct clinical implications for MRD detection. A more sensitive ctDNA platform would enable more accurate identification of patients at high risk of recurrence while sparing low-risk patients from unnecessary treatment. In CRC, numerous studies have reported relatively low concordance between tissue-derived genotyping and plasma ctDNA results in patients with small-volume lung or peritoneal metastases [34,46]. This likely reflects the limited shedding of ctDNA from certain sites of metastasis. Indeed, in the CLM cohort of the GALAXY study, only one-quarter of recurrences in MRD-positive patients occurred outside the liver, whereas approximately two-thirds of recurrences in MRD-negative patients involved the lung or peritoneum. These results show that assay sensitivity may vary according to the site of metastasis.

To address this limitation, efforts have been made to increase sequencing depth and improve the sensitivity of ctDNA detection. Whole-genome sequencing-based (WGS-based) detection of MRD is a promising approach. This method identifies a broad repertoire of tumor-specific variants and tracks a large number of them in plasma, often exceeding 1000 variants. By simultaneously investigating numerous genomic targets, WGS-based assays can provide greater statistical power for detecting low levels of residual ctDNA than conventional non-WGS approaches. Such platforms may therefore overcome the detection threshold limitations of current assays and identify ctDNA that would otherwise remain undetectable. In Japan, the MONSTAR-SCREEN-3 initiative incorporates longitudinal MRD surveillance using an ultrasensitive WGS-based assay. At present, patient accrual has been completed [47].

## 4. Conclusions

In summary, although postoperative chemotherapy has historically improved DFS after resection of CLM, consistent OS benefits have not been demonstrated. This discrepancy highlights the limitations of a uniform treatment approach and underscores the need for more precise risk stratification. The emergence of ctDNA as a marker of MRD provides a promising framework for individualized postoperative management. Growing prospective evidence suggests that ACT may be associated with improved outcomes in patients with ctDNA-positive CLM, while those with ctDNA-negative disease may derive limited benefits. However, these findings should be interpreted with caution, as evidence for treatment-guiding utility remains limited, and further prospective validation is required. Ongoing biomarker-driven clinical trials will be critical in determining whether ctDNA-guided treatment can redefine postoperative management after resection of CLM. At present, ctDNA should be considered a complementary tool rather than a standalone determinant of treatment decisions. Integration with clinical, pathological, and radiological information remains essential to ensure appropriate patient management. Ultimately, integrating MRD detection with clinical decision-making may help bridge the long-standing gap between recurrence risk prediction and meaningful survival improvement in patients with resected CLM.

## Figures and Tables

**Table 1:** Summary of pivotal trials of systemic therapy for resectable CLM.

**Postoperative Adjuvant Chemotherapy Trials**										
**Reference**	**N**	**Median** **Number of** **Lesions**	**Synchronicity**	**Treatment vs. Standard Arm**	**DFS/PFS**	**HR**	***p*** **-Value**	**OS**	**HR**	***p*** **-Value**
Portier et al., 2006 [3]	173	1	28.2%	5-FU/LV vs. surgery alone	5-year DFS: 33.5% vs. 26.7%	0.66	0.028	5-year OS: 51.1% vs. 41.9%	0.73	0.13
Mitry et al., 2008 [4]	278	1	NR	5-FU/LV vs. surgery alone	Median DFS: 27.9 vs. 18.8 months	0.76	0.058	Median OS: 62.2 vs. 47.3 months	0.73	0.13
Ychou et al., 2009 [5]	306	1	NR	FOLFIRI vs. 5-FU/LV	2-year DFS: 50.7% vs. 46.2%	0.89	0.440	3-year OS: 72.7% vs. 71.6%	1.09	0.69
Hasegawa et al., 2016 [7]	180	3	45%	UFT-LV vs. surgery alone	3-year PFS: 38.6% vs. 32.3%	0.56	0.003	5-year OS: 66.1% vs. 66.8%	0.80	0.409
Kanemitsu et al., 2021 [8]	300	1	55%	mFOLFOX6 vs. surgery alone	5-year DFS: 49.8% vs. 38.7%	0.67	0.006	5-year OS: 71.2% vs. 83.1%	1.25	0.42
**Perioperative Chemotherapy Trials**										
**Reference**	**N**	**Median** **Number of** **Lesions**	**Synchronicity**	**Treatment vs. Standard Arm**	**DFS/PFS**	**HR**	***p*** **-Value**	**OS**	**HR**	***p*** **-Value**
Nordlinger et al., 2013 [6]	363	1	35%	Perioperative FOLFOX4 vs. surgery alone	3-year PFS *: 39.0% vs. 29.9%	0.78	0.035	5-year OS **: 51.2% vs. 47.8%	0.88	0.34

5-FU, 5-fluorouracil; ACT, adjuvant chemotherapy; CLM, colorectal liver metastasis; DFS, disease-free survival; FOLFIRI, 5-fluorouracil/folinic acid with irinotecan; FOLFOX, fluorouracil, leucovorin, and oxaliplatin; HR, hazard ratio; LV, leucovorin; mFOLFOX6, modified FOLFOX6; NR, not reported; OS, overall survival; PFS, progression-free survival; UFT, uracil–tegafur. * all eligible patients; ** all randomized patients.

**Table 2:** Summary of studies using ctDNA to detect MRD in patients with CRC.

Reference	Stage	N	Methodology	HR (95% CI) ctDNA−/ctDNA+	RFS Duration	ctDNA− Group (n)	RFS	ctDNA+ Group (n)	RFS
Prognostic studies									
Tie et al. [9]	II	230	Safe-SeqS	18 (7.9–40)	3 years	164	90%	14	0%
Reinert et al. [10]	I–III	130	Signatera^TM^	7.2 (2.7–19.0)	NR				
Tarazona et al. [11]	I–III	150	ddPCR	6.96 (2.57–18.91)	NR				
Tie et al. [12]	III	96	Safe-SeqS	3.8 (2.4–21)	3 years	76	77%	20	30%
Parikh et al. [26]	I–IV	103	Guardant Reveal^TM^	11.2 (NR)	NR				
Henriksen et al. [27]	II/III	839	ddPCR	11.3 (7.8–16.4)	NR				
Nakamura et al. [28]	I–III	333	Guardant Reveal^TM^	16.70 (5.68–49.09)	2 years	291	94.7%	42	40.7%
Nakamura et al. [29]	I–IV	2240	Signatera^TM^	11.99 (10.02–14.35)	3 years	1173	83.5%	336	16.7%
Taieb et al. [30]	III	554	Signatera^TM^	5.75 (4.20–7.87)	2 years	109	88.1%	445	43.5%
ctDNA-guided strategy studies									
Tie et al. [31]	II	455	Safe-SeqS	NR	2 years	302	93.5%	153	92.4%

CI, confidence interval; CRC, colorectal cancer; ctDNA, circulating tumor DNA; ddPCR, droplet digital polymerase chain reaction; HR, hazard ratio; MRD, molecular residual disease; NR, not reported; RFS, recurrence-free survival; Safe-SeqS, safe-sequencing system.

**Table 3:** Available data on ctDNA-based ACT in patients with CLM.

Reference	N	Pre-Operative Chemo-Therapy	Synchronicity	ctDNA+/− (MRD)	DFS (RFS) HR (95% CI), *p*-Value (ACT vs. Observation)		OS HR (95% CI), *p*-Value (ACT vs. Observation)	
					ctDNA+ Group (MRD)	ctDNA− Group (MRD)	ctDNA+ Group (MRD)	ctDNA− Group (MRD)
Kataoka et al. [32]	190	0%	34%	32.1%	0.07 * (0.02–0.26) *p* < 0.001	0.68 * (0.29–1.58) *p* = 0.371	NR	NR
Xu et al. [33]	141	66%	57.4%	33.3%	0.228 ** (0.116–0.446) *p* < 0.0001	HR: NR *p* = 0.39	0.357 ** (0.133–0.963) *p* = 0.034	HR: NR *p* = 0.19

ACT, adjuvant chemotherapy; CI, confidence interval; CLM, colorectal liver metastasis; ctDNA, circulating tumor DNA; DFS, disease-free survival; HR, hazard ratio; MRD, molecular residual disease; NR, not reported; OS, overall survival; RFS, recurrence-free survival. * adjusted HR; ** unadjusted HR.

## Data Availability

Not applicable.

## Abbreviations

ACT	Adjuvant chemotherapy
CAPOX	Capecitabine plus oxaliplatin
CI	Confidence interval
CLM	Colorectal liver metastasis
CRC	Colorectal cancer
CT	Computed tomography
ctDNA	Circulating tumor DNA
DFS	Disease-free survival
FOLFOX	Fluorouracil, leucovorin, and oxaliplatin
HR	Hazard ratio
mFOLFOX6	Modified fluorouracil, leucovorin, and oxaliplatin
MRD	Molecular residual disease
MRI	Magnetic resonance imaging
OS	Overall survival
PFS	Progression-free survival
RFS	Recurrence-free survival
WGS	Whole-genome sequencing
